# Outcomes of gonioscopy-assisted transluminal trabeculotomy in primary congenital glaucoma treatment: a retrospective study

**DOI:** 10.1186/s12886-024-03351-7

**Published:** 2024-02-26

**Authors:** Junyi Lai, Yunsheng Qiao, Chen Tan, Junyi Chen

**Affiliations:** Department of Ophthalmology & Visual Science, Eye & ENT Hospital, Shanghai Medical College, Fudan University, 83 Fenyang Rd, 200031 Shanghai, China

**Keywords:** Gonioscopy-assisted transluminal trabeculotomy, Primary congenital glaucoma, Minimally invasive glaucoma surgery

## Abstract

**Background:**

This retrospective study aimed to evaluate the efficacy and safety of gonioscopy-assisted transluminal trabeculotomy (GATT) in Chinese patients with primary congenital glaucoma (PCG) and identify factors influencing surgical success.

**Methods:**

Fourteen patients (24 eyes) diagnosed with PCG who underwent gonioscopy-assisted transluminal trabeculotomy were recruited, and data on intraocular pressure (IOP), antiglaucoma medication, surgery-related complications, and additional treatments were collected during preoperative and postoperative visits. Surgical success was defined as IOP ≤ 21 mmHg and a reduction of > 30% from baseline, with (partial success) or without (complete success) antiglaucoma medication.

**Results:**

Mean preoperative IOP was 30.41 ± 6.09 mmHg. At the final visit, mean IOP reduction was 16.1 ± 9.1 mmHg (52%), and 19 of 24 eyes were topical medication–free. IOP was significantly decreased at each postoperative visit compared with baseline (*P* < 0.05 for all time points). Cumulative proportions of complete and partial success were 79.2% and 95.8%, respectively, at three years postsurgery. Patients without prior antiglaucoma procedures, without postoperative IOP spikes, and those undergoing complete trabeculotomy exhibited improved surgical prognosis. No permanent vision-threatening complications occurred in the 24 eyes by the end of the respective follow-ups.

**Conclusion:**

Gonioscopy-assisted transluminal trabeculotomy emerged as a safe and effective procedure for PCG treatment, characterized by outstanding IOP reduction efficacy and high surgical success rates.

**Supplementary Information:**

The online version contains supplementary material available at 10.1186/s12886-024-03351-7.

## Background

Childhood glaucoma, a developmental ophthalmopathy occurring at a young age in patients worldwide, leads to irreversible blindness. As the predominant subtype of childhood glaucoma, primary congenital glaucoma (PCG) inflicts severe damage on children’s eyes [1]. Owing to the malformation of the anterior chamber angle and insufficient drainage of aqueous humor, patients with PCG experience elevated intraocular pressure (IOP) [2]. Children with PCG typically present with eye enlargement (buphthalmos), corneal edema and opacification, and Descemet’s membrane ruptures (Haab’s striae) [3]. Furthermore, anterior segment abnormalities, such as iris anterior insertion masking the trabecular meshwork, and an enlarged cup-to-disc ratio in the posterior segment, are identifiable [4].

Angle-based surgery is the primary treatment for PCG, with recent reports suggesting promising outcomes for gonioscopy-assisted transluminal trabeculotomy (GATT) in children with PCG [5–7]. GATT belongs to the category of minimally invasive glaucoma surgeries, encompassing ab-interno, conjunctiva-sparing procedures characterized by moderate IOP reduction efficacy and safety [8]. Assisted by a gonioscope, surgeons use a prolene suture or microcatheter to cleave the trabecular meshwork (TM) behind Schwalbe’s line, creating a “fissure” connecting Schlemm’s canal (SC) to the anterior chamber (AC), thereby facilitating aqueous outflow. In this study, we aimed to evaluate the efficiency and safety of GATT in the eyes of Chinese patients with PCG and identify factors influencing surgical success.

## Methods

A retrospective analysis of patients with PCG who underwent gonioscopy-assisted transluminal trabeculotomy, performed by a single experienced glaucoma specialist (JYC) at Eye and ENT hospital, Fudan University, between July 2019 and July 2023, was conducted. Presurgical and postsurgical data, including IOP at specific time points and topical medications, were recorded.

### Participants

Included patients met the following criteria: (1) clinical signs of PCG evident at 0–3 years of age, (2) IOP > 21 mmHg and more than two ocular features (e.g., increased corneal diameter, corneal edema, Haab’s striae, angle dysgenesis, and glaucomatous optic nerve damage), and (3) an open angle with a visible TM through gonioscopy. The exclusion criteria were as follows: (1) corneal opacity causing blurred views of angle structures, and (2) a history of ocular trauma or secondary glaucoma diagnosis.

### Surgical technique

Following general anesthesia, all patients underwent standard sterile preparation. A 23-gauge corneal paracentesis track (tangentially oriented) was created in either superonasal or inferonasal quadrant, serving as a passage for viscoelastic injection and thermally blunted sutures. A temporal paracentesis was then established. Optimal visualization of the nasal angle, using Swan–Jacob goniolens,, required patients’ heads to be rotated approximately 30° away from the surgeon, and the microscope tilted accordingly. Subsequently, a small goniotomy in the nasal TM was performed using a microsurgical blade through the temporal site. To catheterize and cleave the TM, 5 − 0 or 6 − 0 prolene sutures were used. Briefly, following electrocoagulation-induced thermal blunting of the prolene suture’s tip, a microsurgical forceps was introduced through the temporal paracentesis. The modified tip of the suture was then inserted into the SC through the goniotomy incision. The suture was subsequently advanced 360°. Upon retrieving the distal tip, a complete trabeculotomy was performed by gently pulling the outside end of the suture. Additional attempts were undertaken to ensure maximum cleavage of the TM in cases where the initial trabeculotomy was incomplete (< 360°). These compensatory attempts involved reintroducing the suture into the SC from the original nasal incision in the opposite direction or creating an additional inferior or temporal incision if a 360° trabeculotomy was still not achieved. Finally, an irrigation–aspiration system was used to eliminate any residual viscoelastic substance in the AC.

### Postoperative treatment and follow-up

Patients received topical antibiotics and steroids for 3–4 weeks, tapered at the surgeon’s discretion. They also received 0.5% pilocarpine eyedrops, with gradual dosage reduction over 3 months. Postoperative follow-ups were completed at the first week, first month, third month, sixth month, and thereafter every 3–6 months. At baseline and each postoperative visit, the following data were recorded: IOP, medication use, surgery-related complications, and additional treatments. Regarding methods of IOP measurement, older children (≥ 3 years old) with well cooperation used Goldmann applanation tonometer while younger individuals (< 3 years old) or patients with poor compliance used tonopen tonometer after chloral hydrate administration to record IOP data at preoperative and postoperative visits. Surgical success included IOP ≤ 21 mmHg and a reduction by > 30% from baseline with (partial success) or without (complete success) antiglaucoma medications. Topical steroids and pilocarpine were excluded from antiglaucoma medications under our surgical success criteria. Surgical failures were established if success criteria were not met at two consecutive visits after the first postoperative month or if additional glaucoma surgery was required. Complications were defined as follows: (1) IOP spike, considered IOP ≥ 22 mmHg within the first postoperative month; and (2) hyphema, defined as blood collection in the anterior chamber > 1 mm.

### Statistical analysis

All statistical analyses were performed using SPSS software (version 27.0, IBM Corp.), and data were visualized using GraphPad Prism (version 9.5.1). Significance was set at a 2-tailed P-value of < 0.05. Categorical data were reported as cases and percentages. Normally distributed continuous data (determined via the Shapiro–Wilk test) were presented as means ± standard deviations, whereas non-normally distributed data were represented as medians and interquartile ranges (IQRs). Nonparametric tests were adopted for variables not meeting the parametric test assumptions, e.g., Wilcoxon matched-pairs signed rank tests. Kaplan–Meier survival analysis was used to evaluate cumulative rates of complete or partial success. Log-rank and Breslow–Wilcoxon tests were employed to compare different survival curves.

## Results

Data from this analysis were derived from 24 eyes of 14 patients (13 males and 1 female) with PCG who underwent GATT between July 2019 and July 2023. Demographic and ocular biometric information are detailed in Table 1. The median age at the time of surgery was 1.50 years (IQR: 0.37–4.69). A predominance of male patients were included in this study, aligning with reported PCG characteristics [9, 10]. In 6 of 24 eyes in PCG cases, ocular surgery was performed before GATT. In 10 of 14 patients, bilateral surgery was performed. The mean recorded preoperative IOP was 30.41 ± 6.09 mmHg. In 8 of 24 eyes, antiglaucoma medications were preoperatively administered, with a median of 0 (IQR: 0–1) medications for all 24 eyes. Ocular biometrics, including cup-to-disc ratio (median: 0.9; IQR: 0.7–0.9) and axial length (24.72 ± 2.46 mm), were also collected. The mean length of follow-up was 628.17 ± 377.92 days.

**Table 1:** Baseline demographics and ocular characteristics

	Data Values	No. of Eyes
Age at surgery, y	1.50 (0.37 ~ 4.69)	24
Male sex, %	91.70	22
History of ocular surgery, %	25.00	6
Bilateral GATT, %	83.30	20
Preoperative IOP, mmHg	30.41 ± 6.09	24
No. of preoperative IOP-lowering medications	0 (0 ~ 1)	24
Cup-to-disc ratio	0.9 (0.7 ~ 0.9)	20
Axial length, mm	24.72 ± 2.46	16
Follow-up duration, d	628.17 ± 377.92	24

**Fig. 1:**
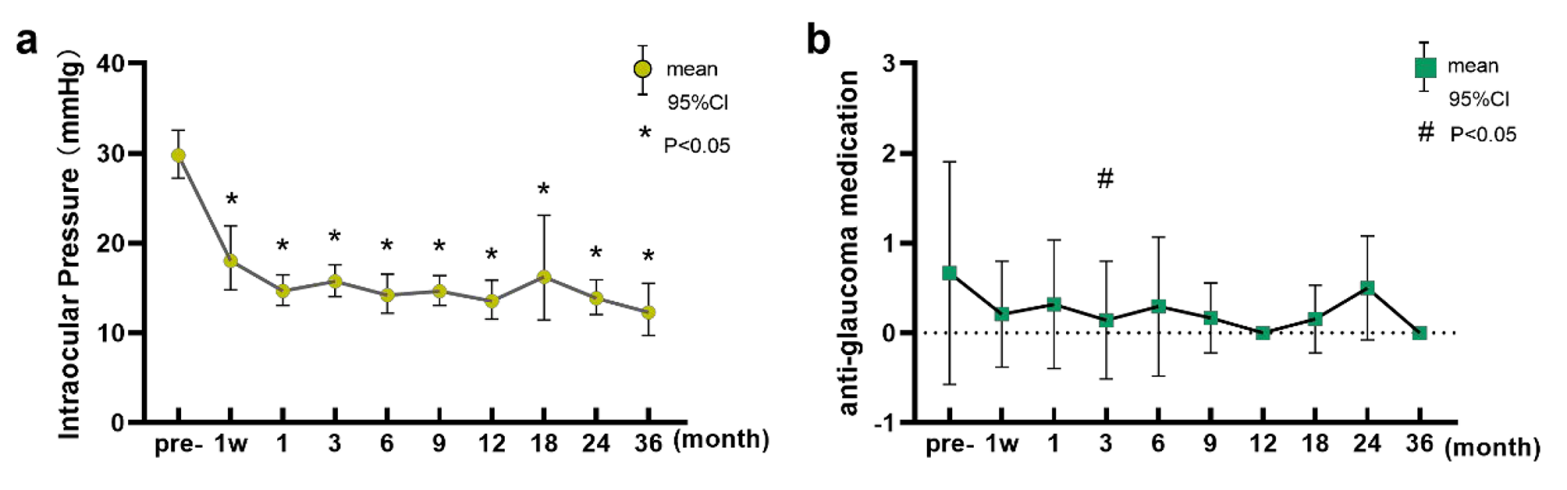
Changes in intraocular pressure (**a**) and antiglaucoma medication (**b**) postoperatively at every follow-up time point

As summarized in Table 2, the mean IOP of all 24 eyes was significantly reduced from baseline at every postoperative time point (1 week and 1, 3, 6, 9, 12, 18, 24, and 36 months; *P* < 0.05 for all). Notably, a discernible trend of medication reduction was observed at most time points (all reducing by ≥ 50%, except at 1, 24, and 36 months). At the last follow-up, effective IOP control (IOP ≤ 21mmHg) was achieved in 23 of 24 eyes (95.8%), and 19 of 24 eyes (79.2%) were topical medication-free. Changes in IOP and antiglaucoma medications over time are shown in Fig. 1.

**Table 2:** Intraocular pressure and medication data obtained through follow-up

Time	intraocular pressure mean(95% CI)					
	n	Preoperative	Postoperative	Reduction (%)	P (compared with baseline)	
1w	23	30.4 (27.7–33.1)	19.8 (16.0-23.7)	10.5 (5.3–15.8) (30)	< 0.001^a^	
1 m	23	30.6 (28.0-33.3)	15.2 (13.4–16.9)	15.5 (12.0–19.0) (48)	< 0.001^b^	
3 m	24	30.4 (27.8–33.0)	16.2 (14.6–17.9)	14.1 (10.7–17.6) (43)	< 0.001^a^	
6 m	17	30.2 (27.1–33.3)	14.7 (12.9–16.6)	15.5 (11.8–19.2) (49)	< 0.001^a^	
9 m	16	29.7 (26.6–32.9)	15.0 (13.3–16.6)	14.8 (11.1–18.5) (48)	< 0.001^a^	
12 m	12	29.6 (26.0-33.1)	13.9 (11.8–16.1)	15.7 (11.6–19.7) (51)	< 0.001^a^	
18 m	8	30.4 (26.9–33.8)	17.6 (10.9–24.4)	12.7 (6.9–18.5) (42)	< 0.001^a^	
24 m	7	32.3 (28.7–35.9)	14.0 (12.1–15.9)	18.3 (13.6–22.9) (56)	< 0.001^a^	
36 m	7	28.7 (21.5–35.9)	12.6 (9.6–15.7)	16.1 (7.7–24.5) (52)	0.003^a^	
medications mean(95% CI)						
1w	24	0.7 (0.1–1.2)	0.2 (0-0.5)	0.5 (-0.1-1.0) (71)	0.08^b^	
1 m	22	0.5 (0.1-1.0)	0.3 (0-0.6)	0.2 (-0.2-0.7) (40)	0.32^b^	
3 m	21	0.7 (0.1–1.3)	0.1 (-0.2-0.4)	0.6 (0-1.1) (86)	0.02^b^	
6 m	17	0.7 (0-1.5)	0.3 (-0.1-0.7)	0.4 (-0.3-1.1) (57)	0.33^b^	
9 m	12	0.5 (-0.2-1.2)	0.2 (-0.1-0.4)	0.3 (-0.5-1.2) (60)	0.48^b^	
12 m	8	1.3 (-0.2-2.7)	0 (0)	1.3 (-0.2-2.7) (100)	0.06^b^	
18 m	13	0.5 (-0.2-1.1)	0.2 (-0.1-0.4)	0.3 (-0.4-1.1) (60)	0.48^b^	
24 m	4	0 (0)	0.5 (-0.4-1.4)	-0.5 (-0.4-1.4)	0.16^b^	
36 m	8	0 (0)	0 (0)	0 (0)	1.0^b^	

CI: confidence interval; n: numbers of patients; ^a^: Paired Student’s t test; ^b^: Paired Wilcoxon signed ranks test

**Fig. 2:**
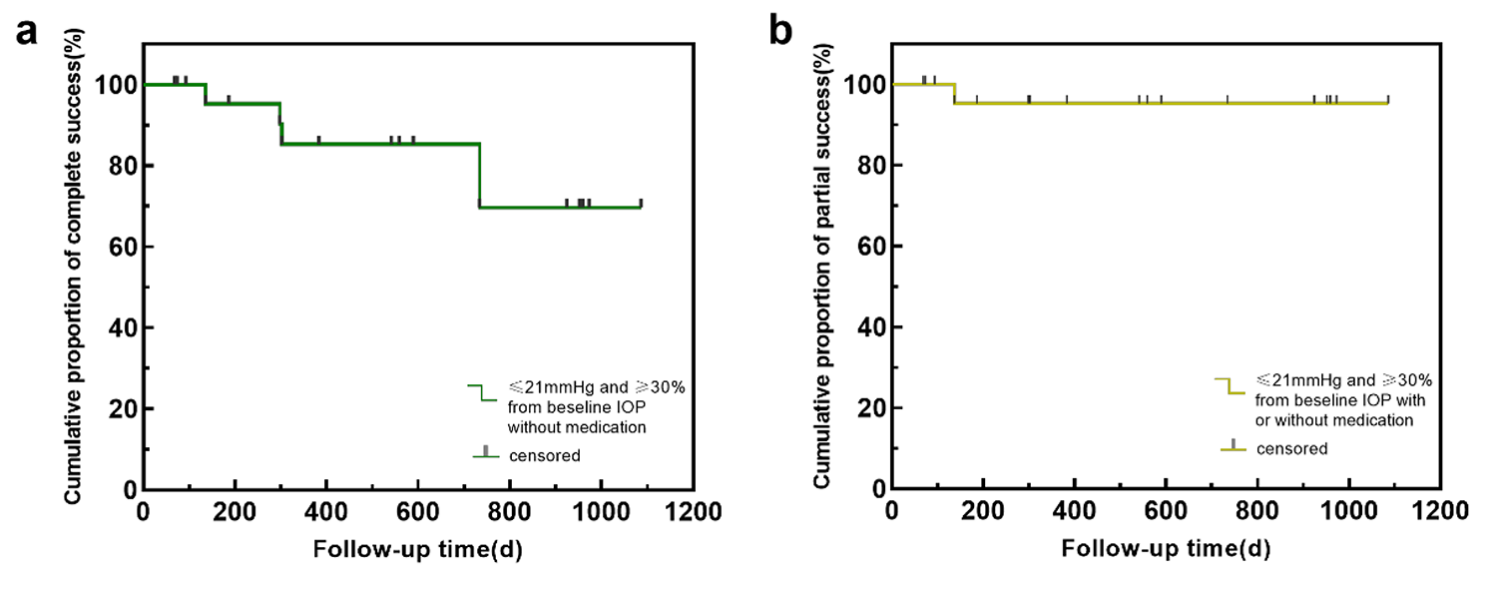
Kaplan–Meier curve showing the cumulative proportions of complete (**a**) and partial (**b**) surgical success over time

**Fig. 3:**
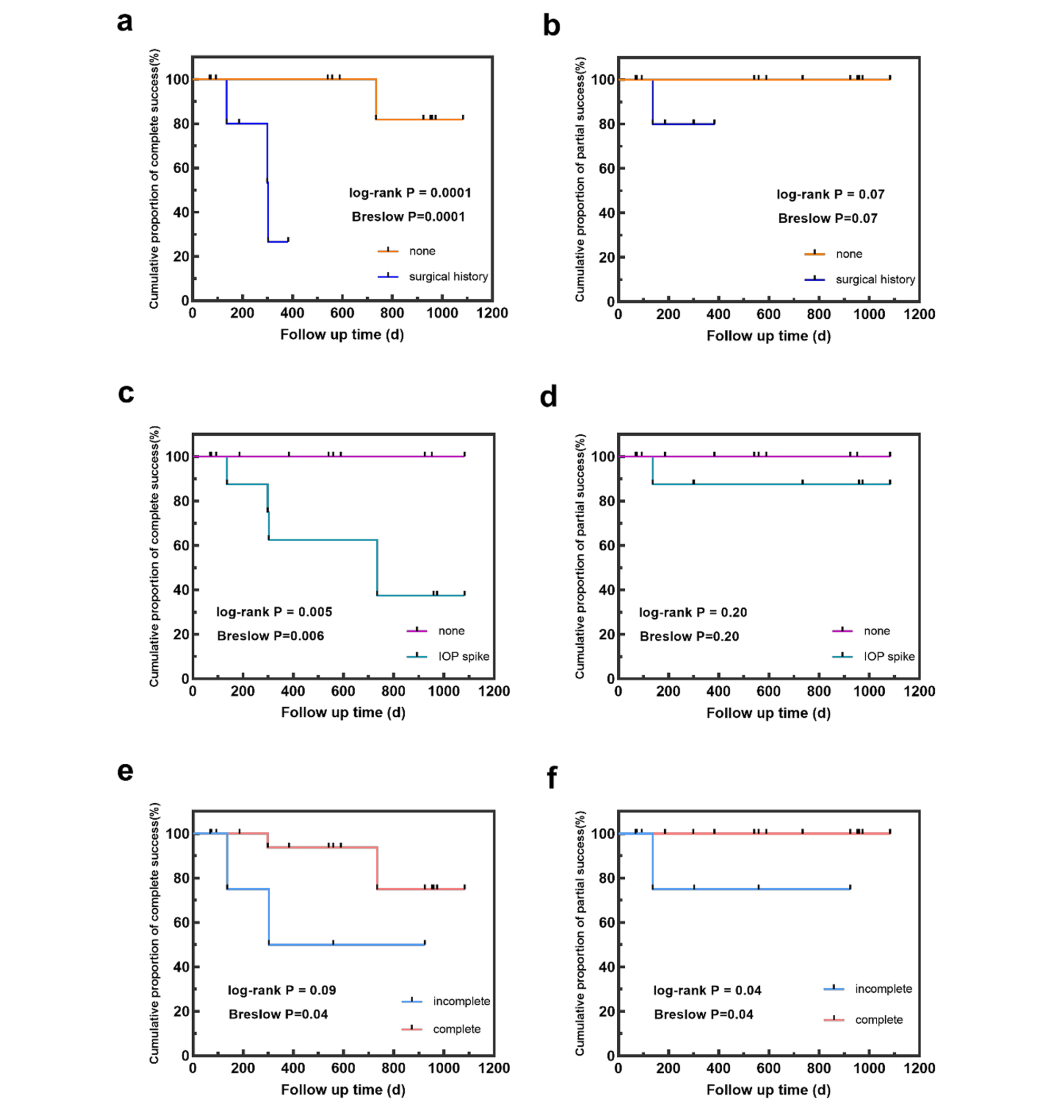
Kaplan–Meier curve of surgical success in GATT group separated based on surgical history [(**a**) complete and (**b**) partial success], IOP spikes [(**c**) complete and (**d**) partial success], and whether the incision was complete circumferential[(**e**) complete and (**f**) partial success]. d, days

The cumulative proportions of complete and partial success were 79.2% and 95.8%, respectively, three years after surgery (Fig. 2). To identify potential factors affecting cumulative surgical success, log-rank and Breslow–Wilcoxon test were applied to compare Kaplan–Meier survival curves based on surgical history (Fig. 3a and b), occurrence of postoperative IOP spike (Fig. 3c and d), and whether the incision was complete circumferential (Fig. 3e and f). For complete success, the differences in survival time distribution between those with and without prior glaucoma surgeries and postoperative IOP spikes were statistically significant (*P* < 0.05 for both Breslow–Wilcoxon and log-rank tests). Eyes in PCG cases with complete circumferential incisions tended to have a better prognosis (*P* < 0.05 for Breslow–Wilcoxon test). For partial success, incomplete TM cleaving decreased the cumulative proportion of surgical success (*P* < 0.05 for both Breslow–Wilcoxon and log-rank test). The aforementioned results are depicted in Fig. 2.

In 10 of 24 eyes that underwent operation, evident intraoperative bleeding and macroscopic hyphema was experienced, all cases of which spontaneously resolved within one month. Transient IOP spike occurred in 10 of 24 eyes, with most (8 of 10) occurring within one week postoperatively. Only 4 of 24 eyes manifested with photophobia, which was gradually alleviated during follow up. Overall, no permanent vision-threatening complications occurred at the end of our 36-month follow-up. Except for changes in antiglaucoma medications, none of our patients required additional treatments.

## Discussion

Trabeculotomy is the preferred solution for primary congenital glaucoma according to the consensus of the World Glaucoma Association [11]. Historically, various ab-externo trabeculotomy techniques using sutures and microcatheters have been employed in patients with PCG. Although these procedures demonstrated effective IOP-lowering efficacy, complications emerged due to conjunctival and scleral dissections [12, 13]. As a representative of newly developed ab-interno procedures, GATT not only maintained comparable IOP control but also mitigated external incision-related complications, such as scarring or leakage [14]. The current study aimed to evaluate short-to-medium term outcomes in Chinese children with PCG who underwent GATT.

Consistent with previous research, GATT proved effective and safe for PCG treatment [6, 15]. Postsurgery, IOPs of our PCG cohort consistently stabilized under 21 mmHg at every follow-up visit (excluding one surgical failure). The mean reduction in patients’ IOP exceeded 40% of baseline at each postoperative time point (except at 1 week). There was a notable trend of reduced medication usage in most cases (reducing by ≥ 50%, except at 1, 24, and 36 months). At the final visit, 19 of 24 eyes were medication-free, although 4 eyes in 2 patients were still receiving 1 antiglaucoma medication for optic nerve protection, as per the surgeon’s discretion. One eye with preoperative lens subluxation, despite receiving three IOP-lowering medications, failed to reach the target IOP, necessitating further surgical intervention. Additionally, our GATT study demonstrated excellent complete and partial success rates, consistent with previous reports [6, 7, 16, 17].

For complete surgical success, children with PCG without prior ocular surgeries tended to exhibit a longer survival time during the 3-year follow up. The impact of previous surgeries on GATT prognosis remains unclear [18–21]. In our cohort, eyes with prior ocular surgery history exhibited an inferior GATT success rate, likely attributed to scarring, inflammation, or fibrotic changes within the TM after previous trabeculectomy (5 eyes) or intraocular environmental disturbance caused by other ocular surgery (one eye with lens subluxation) [20, 22–24].

Similar to previous studies, we examined whether a fully circumferential incision would improve the cumulative success rate [21, 25]. As shown in Fig. 3C, patients with complete trabeculotomies appeared to have a longer survival time than those with incomplete incision under the criteria of complete success, although the log-rank and Breslow test P-values were different. This discrepancy was attributed to the Breslow method giving more weight to deaths/censors at early time points, whereas the log-rank test gives equal weight to all time points. Furthermore, we observed that a 360° incision resulted in a longer survival time in both Breslow and log-rank analyses under the partial success criteria. It is reasonable speculation that fully circumferential incision equates to more open areas for aqueous flow, thereby achieving a more optimal IOP-lowering effect. Nevertheless, there are still conflicting views on the relationship between incision angle and surgical success rate, as well as its possible mechanisms [7, 21, 25]. In this study, we attempted a 360° trabeculotomy in each case. However, 4 eyes without surgical histories failed to be circumferentially incised, which might be attributed to congenital stenosis or transection of Schlemm’s canal. In addition, complete incision could not be achieved in 1 eye with former ab externo trabeculotomy, possibly due to scarring around previous surgical area. These underlying factors may explain the inferior prognosis observed in the incomplete trabeculotomy group.

Eyes in PCG cases without recorded postoperative IOP spikes exhibited a longer survival time in terms of complete success analysis. The mechanisms underlying IOP spikes remain unclear, but numerous studies have highlighted their importance in glaucomatous surgeries [16, 25]. Some suggest that retained viscoelastic material, blood clotting, or resolution of ciliochoroidal detachment may be responsible for early spikes, whereas steroid response may contribute to delayed spikes [26]. In our study, the occurrence of IOP spikes was identified as a factor hindering complete surgical success, consistent with most previous reports [6, 16, 26]. Notably, there may have been some unrecorded IOP spikes after surgeries due to the young age of patients, potentially introducing bias into our analysis.

Several limitations must be considered in our study. First, the retrospective design potentially introduced biases. Second, the small sample size limited the statistical power to evaluate different preoperative and postoperative data. Additionally, we failed to estimate a more extended surgical outcome. Furthermore, although some eyes’ biometric parameters were collected, we could not continue gathering these data or acquire more functional test results owing to the young age of the patients.

In conclusion, to the best of our knowledge, this is the first study focusing on the efficacy and safety of GATT in the eyes of Chinese patients with PCG. Despite our preliminary evidence favoring this procedure, larger cohorts with longer follow-up durations and more consistently divided subgroups are needed to ascertain long-term results regarding GATT as well as risk factors related to surgical failure.

### Electronic supplementary material

Below is the link to the electronic supplementary material.

Supplementary Material 1

Supplementary Material 2

Supplementary Material 3

## Data Availability

Additional file: Supplementary Tables 1–3.

## Abbreviations

GATT: Gonioscopy-assisted transluminal trabeculotomy
PCG: Primary congenital glaucoma
IOP: Intraocular pressure
MIGS: Minimally invasive glaucoma surgeries
TM: Trabecular meshwork (TM)
SC: Schlemm’s canal
AC: Anterior chamber
SD: Standard deviation
CI: Confidence interval

## References

[1] Thau A (2018). New classification system for pediatric glaucoma: implications for clinical care and a research registry. Curr Opin Ophthalmol.

[2] François J (1980). Congenital glaucoma and its inheritance. Ophthalmologica.

[3] Beck AD (2001). Diagnosis and management of pediatric glaucoma. Ophthalmol Clin North Am.

[4] Dietlein TS, Jacobi PC, Krieglstein GK (1996). Assessment of diagnostic criteria in management of infantile glaucoma. An analysis of tonometry, optic disc cup, corneal diameter and axial length. Int Ophthalmol.

[5] Morales J (2013). Current surgical options for the management of pediatric glaucoma. J Ophthalmol.

[6] Qiao Y (2021). Gonioscopy-assisted transluminal trabeculotomy versus goniotomy with Kahook dual blade in patients with uncontrolled juvenile open-angle glaucoma: a retrospective study. BMC Ophthalmol.

[7] Aktas Z (2023). Efficacy and safety of Gonioscopy-assisted transluminal trabeculotomy for primary congenital Glaucoma. J Glaucoma.

[8] Kasahara M, Shoji N (2021). Effectiveness and limitations of minimally invasive glaucoma surgery targeting Schlemm’s canal. Jpn J Ophthalmol.

[9] Alanazi FF (2013). Primary and secondary congenital glaucoma: baseline features from a registry at King Khaled Eye specialist hospital, Riyadh, Saudi Arabia. Am J Ophthalmol.

[10] Liu B (2007). [An investigation on the causes of blindness and low vision of students in blind school in Guangzhou]. Yan Ke Xue Bao.

[11] Chang I, Caprioli J, Ou Y (2017). Surgical Management of Pediatric Glaucoma. Dev Ophthalmol.

[12] Le PH (2021). Ahmed and Baerveldt Drainage Implants in the treatment of Juvenile Open-angle Glaucoma. J Glaucoma.

[13] Luntz MH, Livingston DG (1977). Trabeculotomy ab externo and trabeculectomy in congenital and adult-onset glaucoma. Am J Ophthalmol.

[14] Go MS, Freedman SF (2022). Minimally invasive glaucoma surgery in childhood glaucoma. Curr Opin Ophthalmol.

[15] Shi Y (2022). A prospective study of intraocular pressure spike and failure after Gonioscopy-assisted transluminal trabeculotomy in Juvenile Open-Angle Glaucoma: a prospective study of GATT in JOAG. Am J Ophthalmol.

[16] Wang Y (2021). Outcomes of gonioscopy-assisted transluminal trabeculotomy in juvenile-onset primary open-angle glaucoma. Eye (Lond).

[17] Cubuk MO, Ucgul AY, Unsal E (2020). Gonioscopy-assisted transluminal trabeculotomy as an option after failed trabeculectomy. Int Ophthalmol.

[18] Kono Y (2020). Long-term clinical results of trabectome surgery in patients with open-angle glaucoma. Graefes Arch Clin Exp Ophthalmol.

[19] Grover DS (2017). Outcomes of Gonioscopy-assisted Transluminal Trabeculotomy (GATT) in eyes with prior Incisional Glaucoma surgery. J Glaucoma.

[20] Kuusniemi AM (2020). Ab interno trabeculotomy: key prognostic factors. J Glaucoma.

[21] Quan AV (2022). Factors Associated with Gonioscopy-assisted transluminal trabeculotomy (GATT) complications and failure in children. Am J Ophthalmol.

[22] Tojo N, Abe S, Hayashi A (2017). Factors that influence of trabectome surgery for Glaucoma patients. J Glaucoma.

[23] Capitena Young CE (2018). Histopathologic examination of trabecular meshwork changes after trabecular bypass stent implantation. J Glaucoma.

[24] Lee JY (2014). Morphologic changes in trabecular meshwork after patterned and argon laser trabeculoplasty in cats. Curr Eye Res.

[25] Chen J (2020). Risk factors for complications and failure after Gonioscopy-assisted transluminal trabeculotomy in a young cohort. Ophthalmol Glaucoma.

[26] Quan AV (2020). Gonioscopy-assisted Transluminal Trabeculotomy (GATT) in patients with secondary Open-Angle Glaucoma following vitreoretinal surgery. J Glaucoma.

