# Supplementary material for: Outcomes of gonioscopy-assisted transluminal trabeculotomy in primary congenital glaucoma treatment: a retrospective study
Source: BMC Ophthalmol. 2024 Feb 26;24:88. doi: 10.1186/s12886-024-03351-7 (PMC10898054; doi:10.1186/s12886-024-03351-7)
Supplement: Supplementary file 1 — Supplementary Material 1 [file 12886_2024_3351_MOESM1_ESM.docx]

Supplementary Table 1. Kaplan–Meier survival analysis grouping criteria.

| Variables | Standards |
| --- | --- |
| Previous anti-glaucoma procedures | Yes = 1, No = 0 |
| whether the incision was complete circumferential | Incomplete= 0, complete= 1 |
| Postoperative IOP spike | Yes = 1, No = 0 |
