# Supplementary material for: Outcomes of gonioscopy-assisted transluminal trabeculotomy in primary congenital glaucoma treatment: a retrospective study
Source: BMC Ophthalmol. 2024 Feb 26;24:88. doi: 10.1186/s12886-024-03351-7 (PMC10898054; doi:10.1186/s12886-024-03351-7)
Supplement: Supplementary file 2 — Supplementary Material 2 [file 12886_2024_3351_MOESM2_ESM.docx]

Supplementary Table 2. Log-rank and Breslow–Wilcoxon tests’ results from comparing different subgroups’ complete success rate

| Grouping criteria | Log-rank | |  | Breslow | |
| --- | --- | --- | --- | --- | --- |
|  | χ^2^ | P |  | χ^2^ | P |
| Surgical history | 15.04 | <0.001 |  | 14.4 | <0.001 |
| Whether the incision was complete circumferential | 2.95 | 0.09 |  | 4.07 | 0.04 |
| Post operative IOP spike | 7.96 | 0.005 |  | 7.66 | 0.006 |
