# Supplementary material for: Outcomes of gonioscopy-assisted transluminal trabeculotomy in primary congenital glaucoma treatment: a retrospective study
Source: BMC Ophthalmol. 2024 Feb 26;24:88. doi: 10.1186/s12886-024-03351-7 (PMC10898054; doi:10.1186/s12886-024-03351-7)
Supplement: Supplementary file 3 — Supplementary Material 3 [file 12886_2024_3351_MOESM3_ESM.docx]

Supplementary Table 3. Log-rank and Breslow–Wilcoxon tests’ results from comparing different subgroups’ partial success rate

| Grouping criteria | Log-rank | |  | Breslow | |
| --- | --- | --- | --- | --- | --- |
|  | χ^2^ | P |  | χ^2^ | P |
| Surgical history | 3.2 | 0.07 |  | 3.2 | 0.07 |
| Whether the incision was complete circumferential | 4.25 | 0.04 |  | 4.25 | 0.04 |
| Post operative IOP spike | 1.63 | 0.2 |  | 1.63 | 0.2 |
